# Impact of Elevated Fibroblast Growth Factor 23 (FGF23) on the Cardiovascular System: A Comprehensive Systematic Literature Review

**DOI:** 10.7759/cureus.59820

**Published:** 2024-05-07

**Authors:** Kavya Sai Satya Amaravadi, Poornachandra Nalisetty, Nandini Vadlamani, Sabina Ibrahimli, Farees Ahmad Khan, Jason A Castillo, Sai Sri Penumetcha

**Affiliations:** 1 Internal Medicine, California Institute of Behavioral Neurosciences & Psychology, Fairfield, USA; 2 Internal Medicine, Mamata Medical College, Khammam, IND; 3 Surgery, California Institute of Behavioral Neurosciences & Psychology, Fairfield, USA; 4 Family Medicine, California Institute of Behavioral Neurosciences & Psychology, Fairfield, USA; 5 General Medicine, Chalmeda Anand Rao Institute of Medical Sciences, Karimnagar, IND

**Keywords:** atrial fibrillation, elevated fgf23, etiology and pathogenesis, fgf23, fibroblast growth factor 23, heart failure etiology, left ventricular hypertrophy, molecular biomarker, risk factors cardiovascular diseases, serum biomarkers

## Abstract

Fibroblast growth factors (FGF) are a type of cell signaling proteins that are mostly produced by macrophages. They are essential for a variety of biological activities involved in normal development. Fibroblast growth factor 23 (FGF23) is the newest and youngest member of the FGF endocrine subfamily, along with fibroblast growth factor 19 (FGF19) and fibroblast growth factor 21 (FGF21). In this study, we conduct a systematic review of all known literature to identify the risk of elevated FGF23 in the cardiovascular system. The analysis includes the risk of cardiovascular disease for both primary and secondary causes of elevated FGF23, such as chronic renal insufficiency. This systematic literature review adhered to the Preferred Reporting Items and Meta-Analysis (PRISMA) standards. A total of 4,793 records were identified across different databases. After that, 273 records were retrieved and reviewed. After carefully examining the titles and summaries of each report, 249 additional entries were eliminated. About 24 studies from the remaining records were chosen by primary and secondary authors for screening, and they performed a quality assessment using common quality check tools. Finally, this review included 11 studies. Following a thorough analysis, we came to the conclusion that FGF23 can be regarded as a novel biomarker and should be included in the group of heart biomarkers that have already been identified, such as B-type natriuretic peptide (BNP), for the early identification of a variety of highly prevalent cardiovascular disorders.

## Introduction and background

Fibroblast growth factors (FGF) are a family of cell signaling proteins mainly produced by macrophages. It is a phosphaturic hormone that is primarily released by osteocytes and osteoblasts in bone but is also expressed by salivary glands, the stomach, and, in much lesser amounts, by other tissues such as skeletal muscles, brain, mammary gland, liver, and heart. Fibroblast growth factor 23 (FGF23) is a circulating endocrine hormone that influences mineral metabolism. An increase in FGF23 concentrations occurs as a physiological reaction. They are required for various biological processes involved in normal development. FGF23 is the newest and youngest family member of the FGF endocrine subfamily, along with fibroblast growth factor 19 (FGF19) and fibroblast growth factor 21 (FGF21). In the 20th century, Itoh et al. isolated cDNA from the ventrolateral thalamic nucleus of a mouse and encoded FGF23. FGF23 is a hormonal protein formed by 251 amino acids. It is mainly known for its role in mineral metabolism. In reaction to high levels of 1,25-dihydroxyvitamin D3 (1,25(OH)_2_D_3_) and hyperphosphatemia, osteoblasts and osteocytes generate and release FGF23 [1-5].

Physiological effects of FGF23

**Figure 1:**
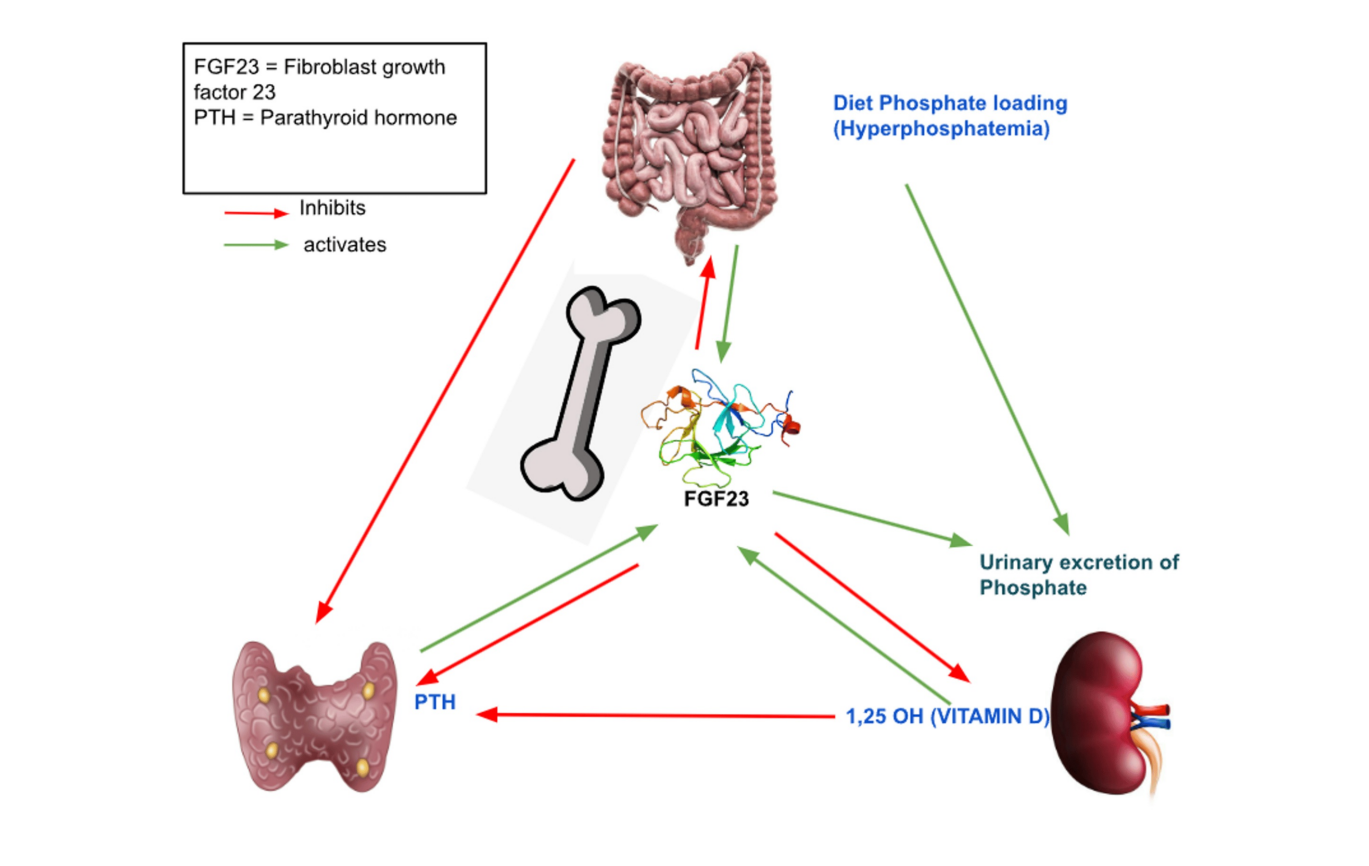
Physiological impacts of fibroblast growth factor 23 (FGF23) Credits: Kavya Sai Satya Amaravadi

The kidneys and parathyroid glands are the most common FGF23 target organs, with membrane-bound Klotho protein acting as an obligatory coreceptor to increase the affinity of FGF receptors (FGFR) to FGF23 [1]. The coreceptor Klotho and FGFR make up the receptor complex that FGF23 binds to in order to exert its effects (referred to as Klotho). Both alternative splicings of the Klotho mRNA and ectodomain shedding of membrane-bound Klotho can produce the soluble version of the Klotho protein. FGF23 binds to the FGFR-Klotho complex, which is found in the distal renal tubule; it inhibits the sodium/phosphate (Na/Pi) cotransporter in the proximal tubule, which could decrease urinary phosphate reabsorption, increase urine excretion, and ultimately lower blood phosphate levels. FGF23 can directly reduce parathyroid hormone (PTH) release and increase PTH via lowering phosphate and vitamin D levels; however, overall PTH levels decline. Additionally, FGF23 suppresses bone mineralization. These key physiological impacts determine and influence the interaction of FGF23 with phosphorus, PTH, vitamin D, and calcium [2]. Figure 1 summarises the physiological impacts of FGF23. 

Elevated FGF23 is linked to adverse cardiovascular changes, including left ventricular hypertrophy (LVH) and vascular calcification. Higher FGF23 levels are associated with increased cardiovascular risk, particularly heart failure-related events rather than events related to atherosclerotic diseases, while the relationship with stroke is less explored due to limited data [3,5].

In the context of chronic kidney disease (CKD), rising FGF23 levels accompany declining kidney function, leading to hyperphosphatemia and cardiovascular complications, including LVH and arterial stiffness. In end-stage renal disease (ESRD), FGF23 levels become exceptionally high. Elevated FGF23 independently predicts cardiovascular issues and can hinder the response to renin-angiotensin-aldosterone system (RAAS) therapy, vital in treating kidney and cardiovascular disease. FGF23-induced imbalances in calcium/phosphate contribute to vascular calcification, a significant factor in cardiovascular problems in both CKD and non-CKD individuals, including those undergoing hemodialysis [6-8].

Maren Leifheit-Nestler et al. found in a case-control study that FGF23 induces cardiomyocyte hypertrophy independently of Klotho via the phospholipase C gamma (PLCγ)/calcineurin/nuclear factor of activated T-cells (NFAT) pathway. In mice, both systemic and intramyocardial FGF23 administration leads to LVH. Significantly, their study revealed that FGF23 specifically triggers FGF receptor 4 (FGFR4) activation in cultured cardiomyocytes, inducing the PLCγ/calcineurin/NFAT signaling pathway in a Klotho-independent manner. Notably, mice deficient in FGFR4 did not manifest LVH in response to elevated FGF23 levels [6]. Recent translational studies have shown that FGF23 directly promotes LVH in mice and hypertrophic development of cardiac myocytes in vitro. The activation of the cardiac FGFR4 mediates the hypertrophic effects of FGF23, which recruits and phosphorylates phospholipase C (PLC), which in turn activates the calcineurin/NFAT signaling cascade, a powerful inducer of ventricular remodeling in response to various pathogenic stimuli [9]. Heart failure stands as the second most prevalent cause of secondary FGF23 overload. Elevated FGF23 levels are observed during exacerbations of HF, demonstrating a correlation with the severity of the disease and serving as a predictor for the risk of HF-related death and hospitalization. Additionally, in the face of acute myocardial injury, cardiac fibroblasts have been shown to produce and release FGF23 [10].

This study conducts a systematic analysis of all existing literature to determine the risk of increased FGF23 on the cardiovascular system. While most studies have primarily focused on the risk of elevated FGF23 in the context of CKD, this particular study provides valuable insights into the direct effects of elevated FGF23 on the cardiovascular system, even in the absence of CKD.

## Review

Method

This systematic literature review was done in accordance with the guidelines laid down in Preferred Reporting Items and Meta-Analysis (PRISMA).

Inclusion/Exclusion Criteria

This study follows strict inclusion and exclusion criteria. Inclusion criteria: study participants aged between 19 years and more than 80 years, studies published from 2015 to 2022, studies in both males and females, only studies in humans, studies in established cardiac disease patients, and CKD. 

Table 1 summarizes the keywords used in the search strategy.

**Table 1:** Keywords used in the search strategy

Concept	Keyword	MeSH strategy
Concept 1	Heart disease, cardiovascular disease, CVD	("Cardiovascular diseases/blood"[Mesh] OR "cardiovascular diseases/diagnosis"[Mesh] OR "cardiovascular diseases/aetiology"[Mesh])
Concept 2	FGF23, fibroblast growth factor 23	Fibroblast growth factor 23 OR Fibroblast growth factor-23/blood"[Majr]

Search Strategy

Cardiovascular disease OR heart disease ("cardiovascular diseases/blood"[Mesh] OR "cardiovascular diseases/diagnosis"[Mesh] OR "cardiovascular diseases/etiology"[Mesh]) AND FGF23 OR fibroblast growth factor 23 OR fibroblast growth factor-23/blood"[Majr].

Study Screening and Selection

**Figure 2:**
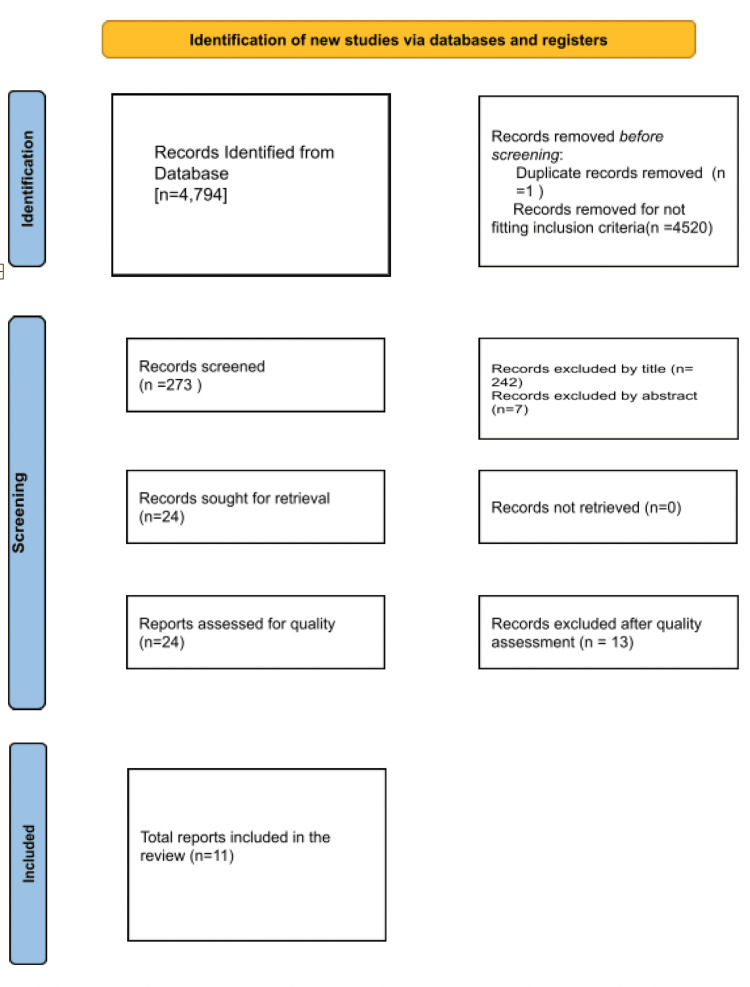
PRISMA flowchart outlining the screening of the data PRISMA: Preferred Reporting Items and Meta-Analysis

The primary author reviewed each article by title and abstract and eliminated the irrelevant results. Both primary and secondary authors further screened the articles for quality appraisal with standardized quality appraisal tools: Cochrane Bias assessment tool for randomized control trials, Newcastle Ottawa tool for non-randomized control trials, and observational studies for quality check. Figure 2 summarizes the PRISMA flow chart which outlines the screening of the data. 

Result

Included Studies

Utilizing the aforementioned search strategy, a total of 4,794 records were identified from various databases. Out of these, one duplicate was eliminated, and 4,520 were excluded for not meeting the study's inclusion criteria. Subsequently, 273 records underwent screening. Following a thorough review of titles and abstracts, 249 records were further excluded. The remaining records, numbering 24, were subjected to quality assessment by primary and secondary authors using standard quality-check tools outlined in the methodology. From this assessment, 13 studies were excluded, resulting in the inclusion of 11 studies in this review.

Study Characteristics 

Table 2 summarises the study type, number of participants in the study, mean age, and study year of all the reports included in the review.

**Table 2:** Summary of the study type, number of participants in the study, mean age, and study year of all the reports included in the review RCT: Randomized controlled trial; ACS: Acute coronary syndrome; CV: Cardiovascular; CKD: Chronic kidney disease; FGF23: Fibroblast growth factor 23; LVH: Left ventricular hypertrophy; STEMI: ST-elevation myocardial infarction; PCI: Percutaneous coronary intervention; MI: Myocardial infarction; AF: Atrial fibrillation; CAD: Coronary artery disease; LVEF: Left ventricular ejection fraction; EPO: Erythropoietin; eGFR: Estimated glomerular filtration rate; CHA2DS2-VASc: Congestive heart failure, hypertension, age, diabetes mellitus, stroke, vascular disease, age, sex; FePi: fractional excretion of phosphate; HEMO study: Hemodialysis study

Study	Year	Study type	Number and characters of participants	Mean age (in years)	Male to female ratio	Inference
Brian A. Bergmark et al. [3]	2018	Secondary analysis of RCT	4947 participants with recent ACS and one additional CV risk	64+/-6	3671:1276	The participants were followed up for a mean of 2.5 years to see the association of FGF23 with recurrent CV events after ACS
Maren Leifheit-Nestler et al. [6]	2016	Retrospective case-control study	24 patients who underwent renal replacement therapy before the age of 15 and are now deceased	11.4+/-8.5	15:9	Detailed autopsy was done to see the association between LVH and expression of FGF23 by cardiac tissue in chronic CKD
Michel Chonchol et al. [7]	2016	Longitudinal study analysis	1340 participants on dialysis for a median 2.7 years	57+/-14	9:11	Over a span of three years, vitamin D and FGF23 levels were regularly assessed to investigate the correlation between low vitamin D and elevated FGF23 levels in the HEMO study, particularly focusing on their impact on infectious and cardiac fatalities
Anne Cornelissen et al. [10]	2021	Observational study	133 participants in intensive care with acute heart failure	66.98+/-12.22	96:37	The participants were followed up for one year to assess the association of elevated FGF23 with a one-year survival rate after acute heart failure
Martin Reindl et al. [11]	2017	Prospective observational study	88 participants with STEMI and treated with PCI	57+/-11	46:11	FGF23 of all participants was measured for four months after MI to assess LV modeling
Winnie Chua et al. [12]	2019	Logistic regression with forwarding selection and machine learning.	638 participants with AF or CHA2DS2-VASc score >= 2	70+/-12	199:147	Baseline data of the participants were used to find blood markers that are associated with AF
MIchele F. Eisenga et al. [13]	2019	Open-label randomized controlled trial	56 individuals with anemia diagnosed with both chronic heart failure and CKD	74+/-6	37:19	Participants were divided into groups and given EPO for 5.7 years to assess the association between EPO and FGF23
Anneke P. Bech et al. [14]	2015	Analysis of an RCT	166 diagnosed with CKD (eGFR 20-70ml/min/1.73m^2^)	53+/-9	56:27	Participants were followed for 4.8 years to assess the impact of FePi on FGF23 and its outcome in CKD
Maren Leifheit-Nestler et al. [15]	2018	Case-control study	24 participants with paraffin-embedded myocardial autopsy samples of the left ventricle received renal replacement therapy before age 15	11.4+/-8.5	15:9	Autopsy samples of the end-stage CKD patients were used to investigate the pro-fibrotic properties of elevated FGF23 in CKD
Brian A. Bergmark et al. [16]	2019	Post hoc analysis of PEACE trial	3,555 patients with stable CAD, LVEF>40%, and serum creatinine<2.0	64+/-7	2882:673	The study participants were followed up for a median of 4.8 years to assess FGF23 and Klotho as biomarkers for CV risk
Masatoshi Miyamura et al. [17]	2015	Retrospective study	831 participants who were admitted to the cardiology unit. Participants on hemodialysis and no atrial fibrillation were excluded	68.6+/-11	625:226	The participants' FGF23 and AF association was analyzed using logistic analysis

The Outcome of the Review Study

The overarching goal of this review article is to investigate the pivotal question of whether blood FGF23 is correlated with cardiovascular morbidity or mortality, either as an independent factor or in conjunction with other blood markers. Additionally, the study aimed to evaluate the potential of FGF23 as a biomarker for predicting long-term morbidity or mortality attributable to cardiovascular diseases. By examining these associations and exploring the interplay with other relevant blood markers, the research endeavors to provide comprehensive insights into the role of FGF23 in cardiovascular health, thereby contributing valuable information for improved risk assessment and personalized healthcare strategies in this domain.

Discussion

This systematic review study focuses on the FGF23 molecule's implications on cardiovascular health. The 11 articles included in this review discuss the impacts of increased FGF23 on the cardiovascular system. Every study included in the review showed either an independent or dependent association of elevated FGF23 with cardiovascular risk. Most of the studies have shown that patients with CKD have a secondary increase in FGF23 due to high phosphate levels, which in turn affects the heart through various mechanisms, as Maren Leifheit-Nestler et al. state in their retrospective case-control study. In both pre-dialysis and dialysis patients, elevated FGF23 levels have also been linked to LVH, which appears to be a possible mechanism explaining the association between higher blood levels of FGF23 and cardiac events [6]. According to Martin Reindl et al.'s prospective case study, individuals without CKD3 and community residents also showed a correlation between FGF23 levels and cardiovascular mortality. Patients with acute or chronic heart failure as well as those with coronary artery disease (CAD) have shown that FGF23 is a potent predictor of cardiovascular mortality [11]. In a randomized controlled trial conducted by Brian A. Bergmark et al. involving a substantial cohort of patients with acute coronary syndrome (ACS), higher FGF23 concentration was associated with an increased risk of severe cardiovascular events, such as cardiovascular mortality or hospitalization due to heart failure. However, there is inconsistent data linking it to atherothrombotic outcomes. Previous studies have established a connection between elevated FGF23 concentration and death, as well as heart failure in non-ACS populations. Among ACS patients, a correlation between FGF23 levels and the likelihood of cardiovascular death, heart failure hospitalization, and all-cause mortality was observed. Additionally, an independent relationship between FGF23 concentration and the likelihood of major adverse cardiovascular events (MACE) was noted, maintaining directional consistency across all components [3]. In the logistic regression investigation conducted by Winnie Chua et al., three biomarkers were identified, specifically elevated B-type natriuretic peptide (BNP) and FGF, showing significant increases in patients with atrial fibrillation (AF) compared to those in sinus rhythm. FGF23, a protein associated with cardiac hypertrophy, chronic renal disease, and vascular stiffness, emerged as a reliable predictor for AF in their study. The research highlights that FGF23 levels are elevated in individuals with AF. Notably, there is no apparent association between FGF23 levels and the duration of AF [12]. In a randomized controlled trial, Michele F. Eisenga et al. underscore the essential role of erythropoietin (EPO) in FGF23 physiology and propose a mechanism linking exogenous EPO use with a higher risk of cardiovascular events, as increased carboxy-terminal fibroblast growth factor 23 (cFGF23) levels were associated with an elevated risk of mortality in the current study [13]. In an observational study, Anne Cornelissen et al. concluded that heart failure is the second most frequent cause of secondary FGF23 excess. FGF23 levels increase during heart failure exacerbations, correlate with disease severity, and predict the risk of heart failure-related death and hospitalization, as well as the likelihood of a therapeutic response to angiotensin-converting enzyme inhibitor (ACE inhibitor) medication, regardless of kidney function [10].

Pathophysiology

In order to determine how FGF23 affects the cardiovascular system, numerous pathophysiologies were shown in the studies that were part of this review. The precise mechanisms linking FGF23 to cardiovascular mortality remain a matter of debate. A significant pathogenetic hypothesis emphasizes indirect alterations to the cardiovascular system caused by simultaneous phosphate excess. However, recent research has unveiled the potential for direct actions of FGF23 on the heart, operating independently of its coreceptor Klotho [11].

FGF23 and Klotho

The renin-angiotensin system (RAS) emerges as a noteworthy mediator of the cardiovascular effects induced by FGF23. Klotho, acting as the coreceptor for FGF23 in both the kidney and vasculature, serves as an inhibitor of RAS. Moreover, FGF23 contributes to the reduction of angiotensin-converting enzyme (ACE) synthesis in the kidneys, which, in turn, acts as a negative regulator within RAS [3]. FGF23 is a phosphaturic hormone derived from the bone that inhibits 1,25(OH)_2_D_3_ production in the kidneys [14].

In the context of CKD, the levels of FGF23 in the bloodstream increase progressively as the glomerular filtration rate (GFR) and the kidney's ability to excrete phosphate decline. This elevation leads to complications such as hyperphosphatemia, insufficient levels of 1,25(OH)_2_D_3_, secondary hyperparathyroidism, and potentially a deficiency of Klotho. In ESRD, FGF23 levels can surge to levels significantly higher than normal. Elevated FGF23 has also been linked to LVH, arterial stiffness, and cardiovascular mortality in CKD patients, both before and after kidney transplantation. FGF23 triggers the RAAS through FGF receptor 1 (FGFR1)/Klotho receptor complexes in the kidney. In this context, it produces classical effects, leading to a decrease in soluble Klotho levels and promoting LVH [6,7,12,15].

When Klotho is suppressed, aldosterone directly initiates the transcription of FGF23 in osteoblastic cells. This suggests that hyperaldosteronism, resulting from Klotho deficiency in CKD, may play a role in the heightened cardiac fibrosis observed. Angiotensin-converting enzyme 2 (ACE2), a vital element of RAS, has been proven to have anti-fibrotic effects on cardiac fibroblasts and to inhibit angiotensin II (AngII)-induced cardiac remodeling. On the flip side, FGF23 acts as a potent inhibitor of ACE2, potentially inducing heart fibrosis and consequent cardiovascular disorders. The elevation of FGF23 and the deficiency of Klotho collaboratively contribute to the activation of the RAS and its well-known adverse downstream effects, including cardiac remodeling and heart failure [15].

The association was once again identified in Brian A. Bergmark et al.'s post hoc study of the Prevention of Events with Angiotensin Converting Enzyme Inhibition (PEACE) trial, specifically when Klotho levels were low rather than elevated. Furthermore, it was demonstrated that the heightened risk associated with low Klotho concentrations was more pronounced in individuals with elevated FGF23 levels. Notably, the therapeutic impact of the ACE inhibitor trandolapril varied based on the presence or absence of Klotho/FGF23. Trandolapril exhibited no discernible effect on individuals with low-risk levels of both Klotho and FGF23. However, it displayed a significant favorable impact on patients with the high-risk combination of low Klotho and high FGF23 concentrations. In the high-risk group (low Klotho and high FGF23), the number needed to treat (NNT) with trandolapril to prevent one cardiovascular death or heart failure hospitalization event was nine, representing a 25% reduction compared to risk stratification based on FGF23 alone (NNT=12). These findings support the hypothesis that a combination of low Klotho and high FGF23 concentrations could serve as an indicator of heightened RAAS activation [16].

Klotho Independent Effects of FGF23

In their retrospective case-control study, Maren Leifheit-Nestler and colleagues suggested that FGF23 specifically activates FGFR4 on cultured cardiomyocytes, initiating PLCγ/calcineurin/NFAT signaling independently of Klotho. Their findings also indicated that mice lacking FGFR4 do not develop LVH in response to increased FGF23 levels. However, the role of the cardiac FGF23/FGFR4 signaling system in the human heart and its potential contribution to LVH development in CKD patients have not been explored until now. This study conducted a comprehensive expression analysis of the cardiac FGF23/FGFR4 system and investigated its association with cardiac remodeling and LVH in a cohort of deceased patients who had experienced childhood-onset ESRD. The gathered data suggested that elevated levels of cardiac FGF23 might have induced LVH through paracrine mechanisms, stimulating FGFR4 and activating the calcineurin-NFAT signaling pathway, particularly in the context of Klotho deficiency, i.e., CKD. The research revealed a decrease in soluble Klotho in the myocardial tissue of CKD patients, showing an inverse relationship with LVH. In contrast, FGF23 had the capacity to bind to and activate FGFR4, both in the presence and absence of soluble Klotho. A recent report by the same researchers demonstrated in cultured cardiomyocytes that FGF23 activated FGFR4 independently of Klotho, mediating pro-hypertrophic effects. Furthermore, the treatment of isolated cardiomyocytes with an FGFR4-specific blocking antibody inhibited FGF23-induced hypertrophy [6].

Moreover, their examination of myocardial tissue from CKD patients indicated an increase in FGFR4 expression alongside diminished levels of soluble Klotho, showing a correlation with cardiac hypertrophy. Conversely, FGFR1 remained unaffected, emphasizing that in CKD, elevated FGF23 levels lead to LVH through Klotho-independent activation of FGFR4. This suggests that elevated cardiac FGF23 levels may drive LVH via paracrine mechanisms, activating the calcineurin-NFAT signaling pathway in the context of Klotho deficiency. Notably, their study uncovered diminished soluble Klotho in CKD patients' myocardial tissue, displaying a negative correlation with LVH, while FGF23 demonstrates the capability to activate FGFR4 irrespective of soluble Klotho presence, as demonstrated in cultured cardiomyocytes, where FGF23-induced hypertrophy was inhibited by an FGFR4-specific blocking antibody [6].

In their observational study, Martin Reindl and colleagues demonstrated that alterations in vascular tissue are contingent on the localized expression of the cofactor Klotho, while FGF23 signaling in cardiomyocytes was reported to be independent of Klotho. The clinical significance of these direct effects of FGF23 is highlighted by their data, revealing FGF23 as an independent predictor of cardiovascular events, even after accounting for abnormalities in mineral metabolism. Consistent with these findings, their data also suggest a direct impact of FGF23 on the myocardium, as there was no observed association between calcium or phosphate levels and left ventricular (LV) remodeling [11].

In their 2018 case-control study, Maren Leifheit-Nestler and collaborators observed a significant enhancement in the expression of genes associated with collagen biosynthesis, including COL3A1 and collagen binding protein 1 (Serpinh1), as well as collagen remodeling genes like Mmp9, Timp2, and Timp3, due to FGF23. This underscored FGF23's involvement in past instances of pathological cardiac remodeling, indicating a profibrotic interplay between cardiac myocytes and fibroblasts. The study also unveiled that active components of the RAAS, namely AngII and aldosterone, strongly induced FGF23, consequently elevating the expression of angiotensinogen (AGT) in both cardiac myocytes and fibroblasts. FGF23, which was expressed and secreted by cardiac myocytes, had a direct impact in the past by stimulating profibrotic factors and inducing fibrosis-related pathways in fibroblasts through a paracrine mechanism. Simultaneously, FGF23 acted on cardiac myocytes, inducing pro-hypertrophic genes and promoting cardiac hypertrophy in an autocrine/paracrine manner. In CKD patients on dialysis, the study observed increased AGT expression, elevated transforming growth factor-beta 1 (TGF-β1), heightened connective tissue growth factor (CTGF), and the accumulation of fibrillar collagens 1 and 3 in the myocardial tissue, all associated with elevated cardiac FGF23 levels and Klotho deficiency. The induction of cardiac FGF23 and reduced Klotho was strongly linked to LVH in the CKD cohort, with mice displaying more severe cardiac hypertrophy and fibrosis under conditions of higher FGF23 and lower Klotho levels. Importantly, Klotho was not expressed in cardiac myocytes or fibroblasts, emphasizing that FGF23's impact on past pathological cardiac remodeling is independent of Klotho. FGF23 also induced oxidative stress and endothelial cell dysfunction, potentially contributing to the progression of cardiovascular diseases in states of Klotho deficiency. Additionally, their data for the first time demonstrated that AGT expression, the primary RAAS component, was enhanced in the past in the myocardial tissue of CKD patients. FGF23 directly induced AGT expression in cardiac myocytes and, to a lesser extent, in fibroblasts, potentially influencing the fibrotic response. This study raised questions about whether enhanced past cardiac expression of FGF23 mediated pathological cardiac hypertrophy through the stimulation of pro-hypertrophic factors such as endothelin 1 (ET1) [15].

FGF23 and AF

According to the logistic regression study conducted by Winnie Chua et al., FGF23 emerges as a key player in myocardial remodeling and cardiac hypertrophy. Not only can FGF23 potentially initiate or exacerbate hypertrophy-related ectopic activity and automaticity, leading to the onset of AF, but it is also associated with endothelial dysfunction. The intricate interplay of these mechanisms, as indicated by Chua et al.'s findings, suggests that elevated levels of FGF23 may contribute, at least in part, to the development of AF in affected individuals [12].

In the current study, we found that FGF23 had a U-shaped association with AF among cardiac patients. This was independent of serum calcium and inorganic phosphorus (iP), and in a subgroup analysis was also independent of intact parathyroid hormone (iPTH) and 25-hydroxyvitamin D (25(OH)D). These findings collectively support the concept that circulating FGF23 may be related to AF, but this raises a question regarding the direct action of FGF23 on AF development. AlphaKlotho, a co-factor of FGF23, is considered to be absent in myocardial cells; therefore, the precise mechanisms by which FGF23 exerts a direct negative cardiac effect remain obscure. FGF23 might exert direct cardiac action via the non-FGF23-FGFR4 axis, but not via the FGF23-FGFR1 axis, which may not require the presence of Klotho. This possibility should be verified in further studies. What is the possible mechanism, if present at all, by which FGF23 facilitates AF development? Ratanapo et al. suggested that FGF23 may play a role in atrial remodeling, which might enhance AF incidence, due to the fact that vitamin D replacement reduced left atrial volume in patients with CKD and that vitamin D decreased FGF23 concentration. In the current study, left atrial dilation (LAD) was greater among patients with higher FGF23, but AF prevalence was the lowest in the third FGF23 quartile. Therefore, whether LAD dilatation intervenes between FGF23 and AF cannot be determined in the current study, and further investigation is needed [17].

Undiscovered Effects of FGF23 Requiring Further Study

Infectious diseases*: *FGF23 has also been suggested to be a regulator of innate immunity. Recent studies reported that FGF23 treatment of mononuclear cells isolated from healthy human peripheral blood and peritoneal dialysis effluents from uremic patients decreased the mRNA expression of CYP27B1. Accordingly, it is reasonable to hypothesize that circulating FGF23 excess decreases the intracrine production of 1,25(OH)_2_D, which consequently decreases the transcription and the production of cathelicidins, leading to an increase in infectious outcomes. These biological effects of FGF23 on monocytic cells support the observation in the present study, which is the first clinical evidence of a relationship between higher serum FGF23 levels and infectious events [7].

Diabetes mellitus: In their observational study, Martin Reindl and colleagues revealed a significant association between diabetes mellitus and FGF23. This finding aligns with earlier studies that have reported a pathophysiological interaction between insulin metabolism and FGF23, especially in patients without CKD [11]. The observed association between diabetes mellitus and FGF23 underscores the importance of future studies to further investigate this relationship and its potential implications.

Exogenous EPO and FGF23: In their randomized controlled trial, Michele F. Eisenga et al. concluded that the administration of exogenous EPO over a 50-week period is associated with elevated levels of cFGF23 that surpass the levels of intact fibroblast growth factor 23 (iFGF23). The baseline cFGF23 levels exhibited a strong correlation with an increased risk of mortality. The observed link between exogenous EPO and cFGF23 levels may signify a potential association between exogenous EPO and adverse outcomes in this patient cohort. To ascertain whether the adverse effects of EPO therapy are indeed linked to its direct impact on cFGF23 levels, further research will be necessary [13].

Does the Sex of an Individual Play a Role in FGF23 Effects?

In their secondary analysis of a randomized controlled trial, Brian A. Bergmark et al. observed that estrogen influences the concentrations of FGF23-modifying compounds, such as PTH, phosphate, and vitamin D24, potentially contributing to the observed sex difference in the association between serum phosphorus concentration and cardiovascular outcomes. Given that the study involved patients following an ACS, most women were expected to be postmenopausal. Menopause is known to lead to increased urine phosphorus retention, likely elevating FGF23 concentration. In fact, postmenopausal women not receiving exogenous estrogen exhibited higher FGF23 concentrations than those receiving estrogen therapy or men. In this case, low FGF23 concentrations could represent either inherently low levels or levels influenced by exogenous estrogen. However, it's plausible that the observed variation by patient sex occurred by chance. Importantly, the predictive value of FGF23 seemed comparable in men and women in the Atherosclerosis Risk in Communities (ARIC) study and Multi‐Ethnic Study of Atherosclerosis (MESA). Nevertheless, this study evaluated the C-terminal fragment of FGF23 instead of the whole peptide quantified in previous research. Therefore, uncertainty exists regarding whether the reported results reflect variations in FGF23 post-translational processing between men and women. Additional research in this area would be beneficial [3].

Applications of FGF23

The clinical management of heart failure patients requires the identification and management of risk factors. To standardize prognosis estimation, several models have been created. These models frequently include a collection of demographic, laboratory, and imaging data that together yield a risk estimation score. However, evaluating every factor that goes into a prognostic model can be difficult and time-consuming, particularly in emergency conditions. However, the evaluation of complex risk scores in emergency situations is sometimes time-consuming and consequently impractical in regular life. The discovery of specific biomarkers that may reliably predict a person's risk may aid in the identification of individuals who require more aggressive therapy and close monitoring. FGF23 may be used as a risk estimation technique that is particularly useful in emergency settings because its levels may be easily determined from the patient's serum [10]. 

FGF23 levels predicted survival one year after hospitalization for acute heart failure in a critically ill patient group with the same accuracy as the Seattle Heart Failure (SHF) model. FGF23 levels and the SHF model can be combined to increase mortality prediction accuracy. The evaluation of FGF23 levels may assist in identifying individuals who require more aggressive treatment, closer monitoring, and follow-up [10]. In a prospective observational study post-ST-elevation myocardial infarction (STEMI), Martin Reindl and colleagues proposed circulating FGF23 as a novel predictor for LV remodeling. Their findings underscore the therapeutic significance of the heart-bone-kidney axis. Notably, the association between FGF23 and LV remodeling was independent of standard prognostic indicators. Moreover, FGF23 did not show a significant correlation with cardiovascular magnetic resonance (CMR) indices of myocardial and microvascular damage or cardiac function. This autonomy from the primary factors influencing postinfarction LV remodeling suggests an additional predictive benefit associated with FGF23 measurements [11].

FGF23 has been demonstrated to be a potent biomarker for the development and progression of CKD, with levels rising even before serum creatinine [10]. Elevated levels of BNP have traditionally served as a well-established marker indicating the presence of AF, while increased concentrations of FGF23 stand out as a novel biomarker strongly correlated with AF. A straightforward assessment that takes into account key factors such as age, gender, body mass index (BMI), BNP, and FGF23 proves to be an effective strategy for identifying individuals with AF. This approach presents an opportunity to enhance the targeted screening of populations through electrocardiogram (ECG) evaluations. Both BNP and FGF23 emerge as valuable tools in stratifying patients with AF, providing valuable insights into their cardiovascular risk profile [12].

FGF23 was likewise found to be a dominant mediator of left ventricular remodeling and hypertrophy in the research included in this review article [7,11].

Distinctive characteristics of cardiomyopathies, namely cardiac fibrosis and LVH, play a significant role in increased cardiovascular mortality, irrespective of the presence of CKD. Additionally, these manifestations of pathological cardiac remodeling are intricately linked [7]. In a follow-up examination of a randomized controlled trial, Brian A. Bergmark and colleagues discovered that among patients enrolled six to 12 months after an ACS, heightened FGF23 concentration was associated with an increased occurrence of a composite outcome, including ischemic episodes, heart failure, and all-cause mortality. Notably, FGF23, a protein indicative of physiological axes distinct from those influencing traditional cardiovascular biomarkers, provided predictive information that added to established clinical predictors and various biomarkers, including estimated glomerular filtration rate (eGFR), BNP, high-sensitivity C-reactive protein (hsCRP), and high-sensitivity troponin I (hsTnI) [3]. As discussed above section, Maren Leifheit-Nestler demonstrated that within cultured cardiomyocytes, FGF23 triggers the activation of FGFR4, irrespective of the presence of Klotho. This activation leads to the mediation of pro-hypertrophic effects. Significantly, the researchers observed that the application of an FGFR4-specific blocking antibody effectively impedes FGF23-induced hypertrophy in isolated cardiomyocytes [6]. In their prospective observational study, Martin Reindl and colleagues pose a crucial question: whether FGF23 acts as the principal mediator of LV remodeling or merely serves as a bystander, reflecting an increased phosphate burden? This distinction requires careful consideration, especially for therapeutic purposes. If FGF23 is validated as the primary inducer of LV remodeling, inhibiting FGF23 could emerge as a viable therapeutic approach for preventing post-STEMI LV remodeling [11].

The limitations of the current study are acknowledged. The current review article covers only research studies published between 2015 and 2022. The age ranges below 19 years and above 80 years are excluded from the review. The study excludes both reviews of the literature and any animal-based studies. In the current article, a limited number of research studies are evaluated.

## Conclusions

FGF23 has both direct and indirect impacts on cardiovascular morbidity and death. Cardiovascular morbidity and mortality are influenced by FGF23 both directly and indirectly. In this review study, we summarized the different negative consequences of increased FGF 23 on the cardiovascular system. A few literature reviews have previously addressed the adverse impacts of FGF23 on the cardiovascular system, although the majority of the studies have focused on secondary effects in CKD patients. In this systematic review, we primarily focused on the direct effects of FGF23 in non-CKD patients. After an in-depth review, we conclude that FGF23 can be considered a novel biomarker and should be added to the list of previously discovered heart biomarkers such as BNP for the early detection of various highly prevalent cardiovascular diseases, particularly given its direct effect on left ventricular remodeling. Additionally, we reviewed the numerous mechanisms of action by which FGF23 exerts its direct effects on the heart, which could serve as an excellent foundation for future research into newer therapeutic drugs. We also evaluated the more recent non-cardiac impacts of FGF23 on the immune system, infectious disorders, and the unintentionally discovered gender-based FGF23 variation that requires more research.
