# Correction: Impact of Elevated Fibroblast Growth Factor 23 (FGF23) on the Cardiovascular System: A Comprehensive Systematic Literature Review 

**DOI:** 10.7759/cureus.c327

**Published:** 2025-09-26

**Authors:** Kavya Sai Satya Amaravadi, Poornachandra Nalisetty, Nandini Vadlamani, Sabina Ibrahimli, Farees Ahmad Khan, Jason A Castillo, Sai Sri Penumetcha

**Affiliations:** 1 Internal Medicine, California Institute of Behavioral Neurosciences & Psychology, Fairfield, USA; 2 Internal Medicine, Mamata Medical College, Khammam, IND; 3 Surgery, California Institute of Behavioral Neurosciences & Psychology, Fairfield, USA; 4 Family Medicine, California Institute of Behavioral Neurosciences & Psychology, Fairfield, USA; 5 General Medicine, Chalmeda Anand Rao Institute of Medical Sciences, Karimnagar, IND

This article has been corrected to specify that 4,794 records were identified, not 4,793. The PRISMA diagram and the paragraph that immediately follows it have been corrected accordingly.

